# Tracheobronchial Foreign Body Aspiration Demonstrating Serial Bronchopulmonary Changes on Computed Tomography

**DOI:** 10.5812/ircmj.18199

**Published:** 2014-05-05

**Authors:** Hidehiro Watanabe, Tomonori Uruma, Gen Tazaki

**Affiliations:** 1 Department of Respiratory Medicine, Tokyo Medical University Ibaraki Medical Center, Ibaraki, Japan; 2 Division of Respiratory Medicine, Tokai University Hachioji Hospital, Tokyo, Japan

**Keywords:** Granuloma, Foreign-Body, Vegetables, Bronchiectasis

## Abstract

**Introduction:**

Tracheobronchial foreign body may often be treated as asthma, chronic bronchitis or etc. especially in patients with no memories of aspiration episodes.

**Case Presentation:**

A 74-year-old woman, suffering from persistent cough, was temporarily misdiagnosed with allergic bronchopulmonary aspergillosis and treated for six months. During this period, computed tomography (CT) findings changed from thickened bronchial walls and a “tree-in-bud” pattern to clubbing bronchiectasis and atelectasis, and no significant bacteria was detected. Finally, a vegetable core was subsequently extracted via flexible bronchofiberscopy. Although the patient's symptoms improved dramatically, the bronchopulmonary lesion remained practically.

**Conclusions:**

We assume that chronologic CT findings of the bronchopulmonary damage by aspiration of a vegetable core, without significant detection of bacteria during the course, will be quite valuable for clinicians.

## 1. Introduction

Tracheobronchial foreign body (TFB) aspiration occasionally occurs in both children and adults (1, 2). However, this is often misdiagnosed as asthma, chronic bronchitis, or recurrent pneumonia, in patients with no alleged recollection of aspiration (3, 4).

Especially, in the case of vegetable containing foods, TFB is not found through a chest radiograph. As a result, a lengthy period is often required before a correct diagnosis of TFB aspiration (5). On computed tomography (CT) scan, TFBs are often difficult to detect depending on their sizes and natures, but secondary bronchopulmonary changes are sometimes visible, suggesting the presence of TFBs. Herein, we reported a patient with tracheobronchial aspiration of a vegetable core, initially diagnosed as having allergic bronchopulmonary aspergillosis (ABPA), based on asthmatic symptoms and characteristic bronchiectatic changes on a CT scan. The initial chest CT findings indicated thickened bronchial walls in the lower respiratory tract and mucus plugging, resulting in a “tree-in-bud” pattern (6), and lacked findings of TFB. Thereafter, findings changed to clubbing bronchiectasis and pulmonary atelectasis, because of mucoid impaction and deterioration. However, the patient's symptoms improved dramatically after TFB extraction via flexible bronchofiberscopy (FBSC) (7). In addition, the clubbing bronchiectasis also partially improved. We hardly found a report on observing the bronchopulmonary changes by TFB as a vegetable core by CT for six months. Moreover, during this period, no significant bacterium was detected. We considered that series of bronchopulmonary damages findings observed by CT were quite valuable.

## 2. Case Presentation

**Figure 1.**
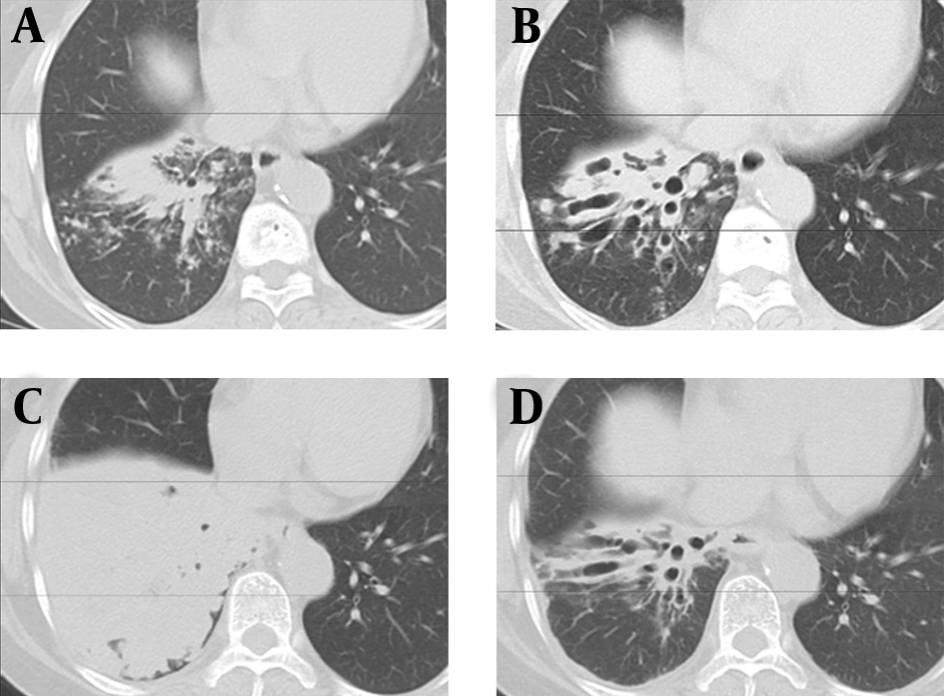
Chest CT Findings Changes Over the Time A) At May 18, 2010 (one month after the first visit), thickened bronchial walls and mucus plugging, causing a “tree-in-bud” pattern, were observed in the right lower lobe. These were considered to be from respiratory tract lesions. B) After four months (September 2), clubbing bronchiectasis was observed. C) After six months (November 18), bronchi of the right lower lobe were further dilated, and mucoid impaction was worsened, while the volume was decreased. D) One month after the TFB removal, clubbing bronchiectasis remained in the right lower lobe, although atelectasis was improved.

**Figure 2.**
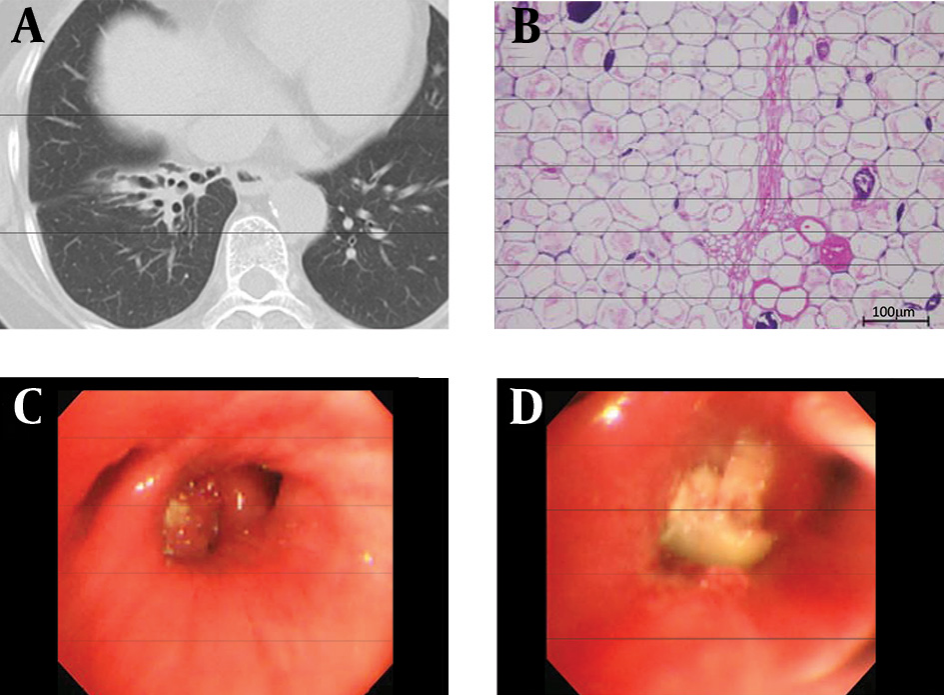
Findings of Chest CT, Bronchoscope, and Pathology of TFB A) Almost 18 months after the TFB removal (May 8, 2012), central bronchiectasis remained in the right lower lobe; however, the check valve effect disappeared, and the peripheral area slightly improved. B) TFB pathological findings: tissue having cell walls, cytoplasm and vessels, was determined to be a hard vegetable core. (H&E staining, magnification × 400). C, D) Bronchoscopic findings during the extraction. C) A white TFB was observed, impacted at B9 in the right lower lobe, surrounded by granulation. D) Removal of the granulation, surrounding a white TFB, which was then extracted.

A 74-year-old Japanese woman, suffering from nonproductive cough for one month, referred to our hospital (a 500-bed city teaching hospital, serving about 560,000 people in southwest Tokyo, Japan) on April 14, 2010, due to having fever for one week. The patient had been medically treated with a calcium blocker and alendronate sodium hydrate because of hypertension and osteoporosis for three years. At the time of the visit for cough, although the patient had neither sputum nor wheezing, there was evidence of only slight infiltration at the lower right lung on the chest X-ray. The patient was almost in the normal range for the ratio of forced expiratory volume in one second (FEV1: 1.42 L) to forced vital capacity (FVC: 1.88 L): the lung function test result was 75.5%, and only normal flora were detected in a provocation sputum culture. She was prescribed a cough medicine and an antibacterial agent (garenoxacin [GRNX], 400 mg/day for five days) with diagnosis of bronchitis; although her cough and other clinical symptoms had reduced for several days, they subsequently returned to their original levels. She referred to our hospital again on May 18 (one month after the first consultation). On the reconsultation day, the patient’s body temperature was 37°C and her unproductive cough was worsened. There was no evidence of swollen lymph nodes, eczema or other abnormalities. The patient’s white blood cell (WBC) count was 11000/µL, the percentages of neutrophils and eosinophils were 69.3% and 2.7%, respectively, and C-reactive protein (CRP) concentration was 1.25 mg/dL (Table 1). No other abnormalities were observed in the blood examination. However, a chest CT showed evidence of thickened bronchial walls and mucus plugging, causing a “tree-in-bud” pattern on the right lower lobe (Figure 1A). At that time, we considered the patient temporarily diagnosed with a bronchial hypersensitivity, such as ABPA, that the imaging findings suggested. However, her unproductive cough remained dry, and no significant bacteria or fungus was detected in cultures of provocation sputum and gastric juice. In addition, both *Aspergillus* antigen and IgE antibody were negative, and a T-SPOT.TB test was also negative. The patient was treated with cough medicine, an inhaler (budesonid/formoterol), and a leukotriene antagonist after a short-term GRNX prescription (400 mg/day for five days). Although there was no episode of fever, the patient’s unproductive cough was maintained, and the CT findings continued to change from thickened bronchial walls to increased clubbing bronchiectasis and mucoid impaction (Figure 1B). Treatment with a macrolide (clarithromycin [CAM], 400 mg/day) was then added, after the patient’s unproductive cough became productive in September 30. Meanwhile, there were no eosinophils in the provocation sputum, and no significant bacteria or fungi were detected in the accompanying cultures. These findings suggested that the patient was suffering from neither bronchial asthma nor ABPA. Her clinical symptoms maintained with scant improvement, and a high fever of 39°C and right chest pain appeared under productive cough and sputum on November 18. The patient was admitted to our hospital for pneumonopleuritis at that time with a respiratory rate of 20 breaths/min, blood pressure of 142/88 mmHg, regular pulse of 96 beats/min, and an oxygen saturation of 95% on room air. The patient’s WBC count was 22500/µL, the neutrophils and eosinophils percentages were 82.0% and 0.0%, respectively, and CRP was at 26.29 mg/dL. Since a strong inflammatory response was observed, we considered that the patient had bacteremia. The results of other blood examinations were also in the normal ranges, including the transaminases, except for lactate dehydrogenase (LDH), which was slightly elevated (243 U/L) (Table 1). Both pneumococcal and legionella urine antigen rapid tests were also negative, and no pathogenic organism was detected, although hemoculture, sputum culture, and urinary culture were performed along with antibacterial agent (cefpirome sulfate [CPR], 4 g/day, drip infusion) preinitiation treatment. Four days later, after treatment with CPR, the patient’s fever abated, although a part of her productive cough continued, and WBC and CRP results improved to 12700/µL and 8.78 mg/dL, respectively. On November 25, we considered that FBSC should be performed, as the clubbing bronchiectasis and pulmonary atelectasis at the right lower lung had not improvements (Figure 1C). FBSC findings of rubor and swelling of the bronchial mucosa at the distal intermediate bronchus were observed. When the purulent sputum was absorbed carefully, a granuloma-like structure was observed, surrounding a white foreign body at the right B9 bronchus (Figure 2C), and a roughly 8 × 5 × 2 mm^3^ white foreign body was then extracted from that location (Figure 2D). As a result of a pathology search, this white foreign body was identified as a vegetable core (Figure 2B), along with a bronchus necrotic tissue. In the culture of the bronchial lavage, no significant bacteria were detected, except for scant alpha *streptococcus*. After extraction of the foreign body, the productive cough and sputum, as well as the clinical symptoms and blood examination results improved dramatically, and the pulmonary atelectasis also improved (Figure 1D). The patient left our hospital on November 30. The cough had disappeared completely during the 18-month period following discharge. As for the chest CT findings at May 8, 2012 (almost 18 months after the TFB extraction), the peripheral portion of the clubbing bronchiectasis observed in the right lower lung had improved, although the central portion remained (Figure 2A) .

**Table 1.** Laboratory Findings in the Clinical Course ^a, b^

	2010			
	May 18	September 30	November 18	November 30
**Hematology**				
WBC, /mL	11000	7700	22500	7500
Seg, %	69.3	69	82	68.6
Eosino, %	2.7	3.2	0	3.8
Mono, %	5.6	7.5	9	4.4
Lymp, %	21.6	20.2	3	21.7
RBC, × 10^4^/mL	441	400	363	355
Hb, g/dL	12.3	11.1	10.3	9.9
Ht, %	39.2	35.5	31.8	32.5
Plt, × 10^4^/mL	41.7	43.2	76.1	61.5
**Biochemistry**				
BUN, mg/dL	11	15	10	12
Cr, mg/dL	0.5	0.63	0.89	0.48
AST, IU/L	22	14	28	15
ALT, IU/L	14	9	30	12
LDH, IU/L	155	180	243	138
Glu, mg/dL	99	118	238	100
CRP, mg/dL	1.25	0.40	26.29	0.14
**Infection**				
Sputum Culture	NF	NF	NF	NF
*Aspergillus* Ag, CI	0.3	ND	0.3	ND
*Aspergillus* Ab IgE, UA/mL	> 0.34	ND	ND	ND
β-D glucan, pg/mL	> 5.0	ND	> 5.0	ND
T-SPOT.TB	(-)	ND	ND	ND
**Urine antigen test**				
*S. pneumoniae*	ND	ND	(-)	ND
*L. peumophila*	ND	ND	(-)	ND

^a^ Abbreviations: ALT, alanine transaminase; AST, aspartate transaminase; BUN, blood urine nitrogen; CI, confidence interval; CRP, C-reactive protein; LDH, lactate dehydrogenase; NF, normal flora; ND, note done; RBC, red blood cell; WBC, white blood cell.

^b^ Urine antigen tests (Binax NOW, Alere, California USA).

## 3. Discussion

We reported a case of vegetable core aspiration in a healthy 74-year-old woman, presenting chronic cough and recurrent fever, who was ultimately diagnosed with TFB by FBSC, six months after her initial visit to the medical institution. The interesting point was that serial CT scans of the chest revealed various bronchopulmonary lesions, changing before and after the TFB extraction. TFB aspiration is much more prevalent in children than in adults. In a large-scale pediatric report, 68% to 75% of cases occurred in children younger than 3 - 5 years (8, 9). The TFB site was right bronchus in 54.6% of cases (8) and left bronchus in 53.6% of them (9), with a nearly balanced right/left ratio. Most were discovered via image analysis (80.3%), and roughly 20% were normal findings. Extraction by bronchoscopy under general anesthesia was effective, and most were removed in this manner (99.72%) (8).

On the other hand, TFB aspiration is rare in adults, comprising roughly 0.33% of bronchoscopy cases (7). Ninety percent of patients have a risk factor for aspiration, with stroke being the most common of these, at 30%. Neurological and neuromuscular diseases were involved in 58% of all cases. TFB was suggested by medical history in 38.4%. However, it was discovered by chest X-ray in only 7% of patients. The site was usually the right side (75.6%), and the most common TFBs were animal and fish bones (39.5%). In 90.7% of the patients, TFBs were successfully removed under FBSC, whereas in 8.1%, TFB was extracted with a flexible bronchoscope through an endotracheal tube.

CT is useful for TFB discovery (1, 10). Zissin, et al. (10) examined 19 cases of TFB (11 males and 8 females, 26-89 years) and reported that CT findings in the affected lobe were volume loss, hyperlucency with air trapping, and bronchiectasis. However, as in this case, foreign bodies other than metal, teeth, dentures, and bridges are often difficult to detect by CT (7). Thus, in adults as in children, TFB is often misdiagnosed and treated as chronic bronchitis, recurrent pneumonia, persistent cough, or asthma (3, 4). In this case, the serial structural changes in the lung due to the foreign body were observed over time via CT. At the initial CT scan, an inflammatory reaction to the TFB impacted in the right B9 bronchus occurred, and a “tree-in-bud” pattern due to thickening of the peripheral bronchial wall and mucus plugging was observed (Figure 1A), resembling ABPA-like respiratory lesions (6). At this time, we suspected to ABPA, because persistent cough and asthma-like symptoms were also observed. After four months, the structure changed to clubbing bronchiectasis with mucoid impaction, suggesting that granulation was formed to cover the TFB, probably serving as a check valve (Figure 1B). After six months, when FBSC was performed, the right B9 bronchus and entire right lower lobar branch were occluded with granulation tissue covering the TFB (Figure 2C). A CT scan revealed that pulmonary atelectasis was developed in the lower right lobe with mucoid impaction (Figure 1C). Concomitant with this development, the clinical symptoms (hyperthermia and productive cough) worsened. However, the pulmonary atelectasis was improved by removal of the foreign body under FBSC, although the clubbing bronchiectasis remained (Figure 1D). The patient’s clinical symptoms improved dramatically. In a follow-up CT almost 18 months after the removal, bronchiectasis remained central, but had improved peripherally (Figure 2A).

Involving a small foreign body composed of vegetable matter, discovery is difficult when there is no patient recollection of aspiration. In the initial phase, the TFB brought about the CT findings of "tree-in-bud" pattern. Those imaging and clinical findings caused the temporary misdiagnosis. Subsequently, it changed to mucoid impaction and bronchiectasis. We considered that series of bronchopulmonary damage findings observed by CT, such as this case, can be quite valuable and instructive for clinicians. Even if significant bacteria cannot be detected, it is important to keep track of changes in CT images in the cases of treatment-resistant persistent coughs. If mucoid impaction or bronchiectasis is observed by CT, FBSC or virtual navigation should be actively performed.
